# Anesthetic Management of a Giant Thymolipoma Causing Near Total Lung Collapse: A Case Report

**DOI:** 10.7759/cureus.97795

**Published:** 2025-11-25

**Authors:** Monika Yadav, Sudhir Kumar, Sangeeta Khanna, Jyotirmoy Das, Mohan V Pulle

**Affiliations:** 1 Anesthesiology and Critical Care, Medanta - The Medicity, Gurugram, IND; 2 Thoracic Surgery, Medanta - The Medicity, Gurugram, IND

**Keywords:** airway compression, anterior mediastinal mass, double-lumen endobronchial tube, giant thymolipoma tumour, post-operative pulmonary complications (ppcs)

## Abstract

Thymolipoma is a rare benign anterior mediastinal tumour. Although often clinically silent, it can lead to significant pressure effects on the airway, mediastinal structures and great vessels. Anaesthesia induction and the use of muscle relaxants in such patients may precipitate airway collapse and difficult bag-mask ventilation due to the size, location and weight of the mass. Haemodynamic instability, arrhythmias and injury to vital structures contribute to the perioperative challenge. Major postoperative concerns include pain management and reduction of postoperative pulmonary complications (PPC), including re-expansion pulmonary edema. We report a case of massive anterior mediastinal thymolipoma . The growth caused marked mediastinal displacement to the right, compression of the left main bronchus, complete collapse of the left lung and partial collapse of the right lung. Despite this, the patient remained asymptomatic at rest, maintaining an oxygen saturation of 89% on room air. Anesthetic management involved controlled induction rather than awake intubation, reflecting a preparedness-based strategy with extracorporeal membrane oxygenation and rigid bronchoscopy available on standby, guided by the patient’s clinical stability and radiological assessment, rather than an avoidance-based approach.

## Introduction

Thymolipoma is an uncommon benign tumor of the anterior mediastinum, composed of mature adipose tissue and thymic elements [1]. The consistency is soft and pliable, and as the tumor enlarges, it often expands inferiorly, fitting into anatomical spaces between the lungs, heart and mediastinal structures. On frontal chest radiographs, these masses may mimic cardiomegaly or massive pleural effusions. As thymolipomas frequently remain asymptomatic until considerable mass effect occurs, they may grow to large sizes, occasionally weighing multiple kilograms [2]. While typically asymptomatic, large thymolipomas may cause compressive symptoms from displacement of adjacent intrathoracic structures, including cough, dyspnea, chest discomfort and hemoptysis. An association with autoimmune conditions such as myasthenia gravis, Graves’ disease and aplastic anemia has also been reported [3]. In contrast, other anterior mediastinal tumors such as thymomas and lymphomas tend to present earlier in their course, often producing more acute compressive symptoms due to their firmer consistency and invasive growth patterns. Surgical excision remains the treatment of choice, being both diagnostic and curative, with no documented risk of recurrence or malignant transformation on long-term follow-up [4]. We present a case of a giant thymolipoma occupying almost the entire thoracic cavity, with significant airway and lung compression yet minimal clinical symptoms. This report highlights the successful controlled induction in a patient with near total lung collapse with minimal symptoms, anesthetic challenges, particularly relating to airway management and ventilation, and emphasises the importance of meticulous perioperative planning.

## Case presentation

**Figure 1:**
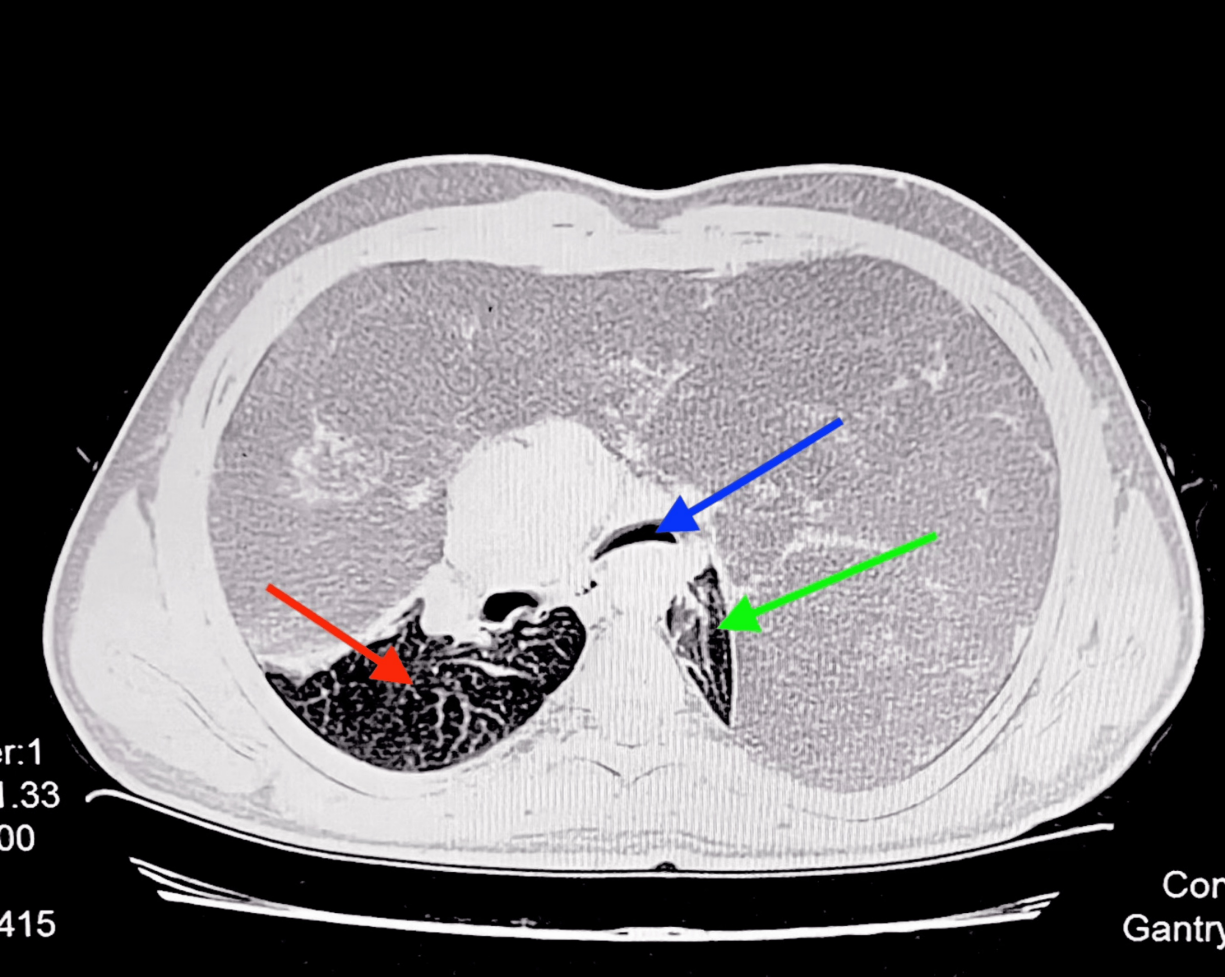
CECT thorax axial section showing huge heterogenous mass occupying bilateral thoracic cavities and right mediastinal shift. Red and green arrows: compressed right and left lung, respectively; Blue arrow: compressed left main bronchus.

A 31-year-old male patient, weighing 70 kg, presented with a four year history of chest pain and gradually progressive dyspnea consistent with Modified Medical Research Council (MMRC) grade I, and was able to climb two flights of stairs, albeit with some limitation. The patient remained asymptomatic at rest and tolerated the supine position without discomfort. There was no history of orthopnea, positional dyspnea, daytime sleepiness, cough, hemoptysis, fever or significant weight loss. The patient denied any symptoms suggestive of superior vena cava syndrome. His past medical history was significant for well-controlled diabetes mellitus (HbA1C 6.8%). A contrast-enhanced computed tomography (CECT) scan of the chest showed a large heterogenous predominantly fatty attenuation lesion seen in bilateral thoracic cavities measuring approximately 29 × 25.5 × 29 cm. The mass resulted in near-complete collapse of the left lung, narrowing of the left main stem bronchus and partial collapse of the right lung, with a marked rightward mediastinal shift (Figure 1).

The ultrasound-guided core needle biopsy was inconclusive, as the predominantly fatty composition and scant thymic elements characteristic of thymolipomas limit the diagnostic adequacy of small tissue samples. A definitive diagnosis was achieved only following surgical excision and histopathologic evaluation of the resected specimen.

**Figure 2:**
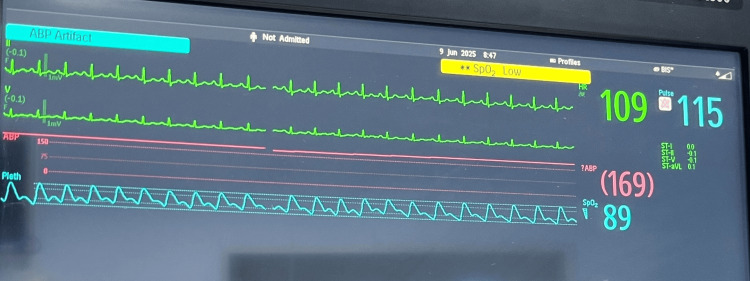
Monitor showing baseline peripheral oxygen saturation.

On presentation, his peripheral oxygen saturation was 89% on room air (Figure 2).

Auscultation revealed diminished breath sounds on the left side of the chest. A comprehensive pre-anesthetic assessment was performed as per institutional protocol. Arterial blood gas analysis (ABG) showed a PaO₂ of 62 mmHg and a PaCO₂ of 50 mmHg. A transthoracic echocardiogram demonstrated a normal left ventricular ejection fraction and right ventricular systolic pressure (RVSP) of 28 mmHg. The patient was accepted for surgery with an American Society of Anesthesiologist physical status (ASA-PS) Classification II.

Informed and written high-risk consent was obtained. A thoracic epidural catheter was placed at the T6-T7 interspace for perioperative analgesia. The patient fasted for six hours and received no sedative premedication. A rigid bronchoscope was kept readily available in the operating room (OR) in anticipation of potential central airway collapse. An extracorporeal membrane oxygenation (ECMO) system was kept on standby along with intravenous sugammadex in case of emergency airway or ventilation failure. Standard ASA monitors were applied, and two wide-bore intravenous cannulas were secured (including one in the left saphenous vein). For invasive blood pressure monitoring, left radial artery cannulation was performed under local anesthesia. Both groins were prepared and draped in a sterile manner to allow immediate cannulation if ECMO initiation became necessary (left side for central venous catheter and sheath; right side for ECMO cannulas).

Fentanyl 1 mcg/kg was administered intravenously and pre-oxygenation was carried out in the semi-upright position using 5 cmH₂O pressure-support ventilation for five minutes. Anesthesia was induced with a titrated dose of propofol (80 mg). After confirming adequate bag-mask ventilation, rocuronium (100 mg) was administered for neuromuscular blockade. Because of distorted airway anatomy and compression of the left main bronchus, a right-sided double-lumen tube , 37 French (Romsons® , India ) was selected and inserted under fibreoptic bronchoscopy guidance (AMBU® aScope^TM^ 4 Broncho SLIM 3.8 mm outer diameter, Denmark)

**Figure 3:**
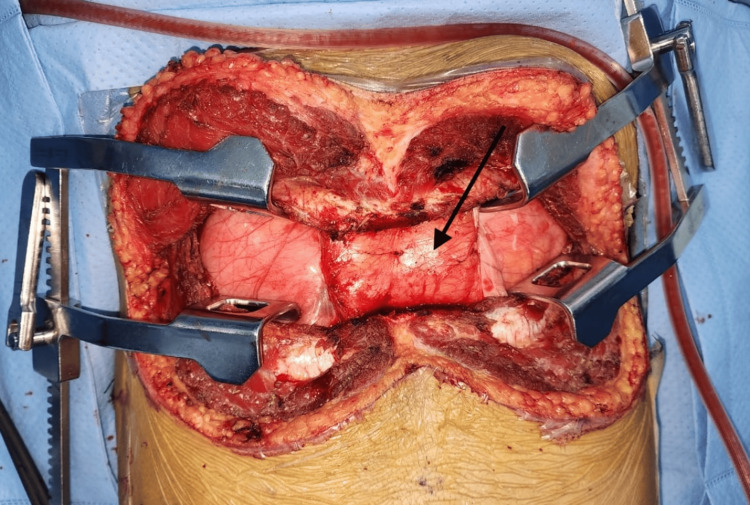
Thymolipoma tumor mass (Black arrow) visualized through a clamshell incision

Anesthesia was maintained with a 50:50 mixture of oxygen and air, and sevoflurane at a minimum alveolar concentration (MAC) of 0.7-0.8. The mediastinal mass was approached via a clamshell incision, corresponding to dermatomal level T3-T6 (Figure 3).

**Figure 4:**
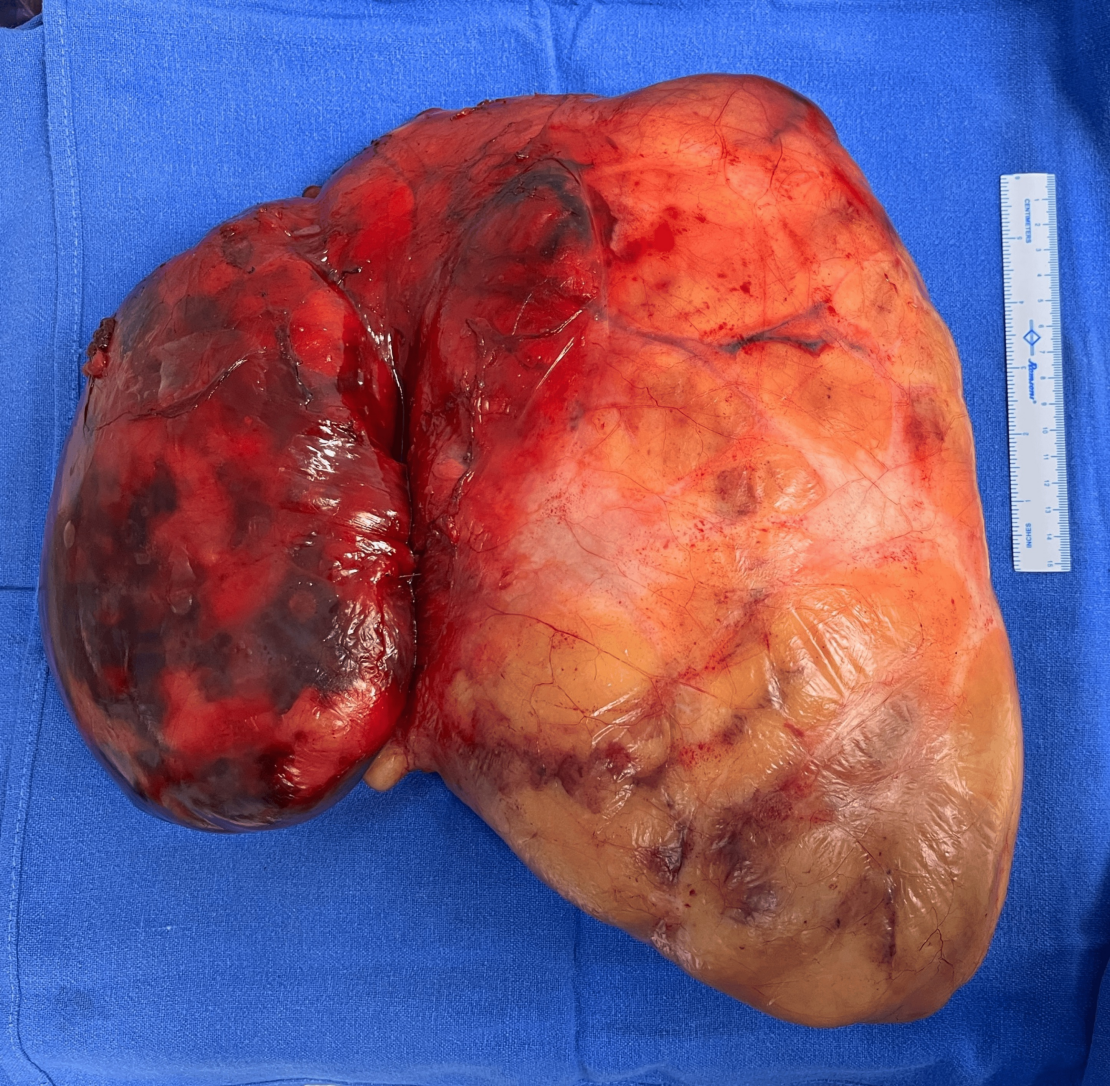
Resected specimen compared with a 15 cm scale

Intraoperatively, frequent adjustments of the double-lumen tube were required due to anatomical distortion and surgical manipulation. As anticipated, ventilation required high airway pressures; therefore, pressure-controlled ventilation with volume guarantee (PCV-VG) mode ( tidal volume 6-7 ml per kg predicted body weight, positive end expiratory pressure (PEEP) 5-8cmH_2_O, plateau pressure less than 30cmH_2_O) was employed to ensure lung-protective ventilation. The surgical procedure proceeded uneventfully, with no significant blood loss or cardiorespiratory instability, and lasted approximately six hours. The excised mass measured approximately 30 × 30 cm and weighed 5.1 kg (Figure 4).

After tumor excision, the collapsed left lung was gradually re-expanded using sustained inflation at 25 cmH₂O while avoiding excessive manual ventilation to minimise the risk of re-expansion pulmonary oedema. Postoperative analgesia was initiated via epidural infusion of 0.1% ropivacaine with fentanyl (2 mcg/mL) at 6 mL per hour. The patient was successfully extubated on the operating table and transferred to the intensive care unit (ICU) for further monitoring and care. Postoperative analgesia was provided using a continuous epidural infusion supplemented with intravenous acetaminophen and non-steroidal anti-inflammatory drugs (NSAIDs) as part of a multimodal pain-management strategy. The patient did not experience any postoperative complications and was discharged successfully.

## Discussion

This case underscores the considerable anesthetic challenges posed by large anterior mediastinal masses, particularly those involving airway compression. Although thymolipomas are benign and often asymptomatic, they can attain enormous size and produce significant cardiopulmonary compromise. Clinical manifestations typically arise only after substantial compression of the tracheobronchial tree or great vessels. The prevailing recommendation in such cases has been to maintain spontaneous ventilation during induction, as administration of muscle relaxants may precipitate airway collapse secondary to loss of airway tone [5]. Accordingly, awake intubation and avoidance of neuromuscular blockade are frequently advocated to preserve spontaneous respiration and prevent catastrophic obstruction. In recent years, high-flow nasal oxygen (HFNO) has also emerged as a useful adjunct during spontaneous ventilation, providing improved oxygenation and apneic reserve without compromising airway patency. This technique can enhance safety during induction in patients at risk of dynamic airway collapse.

However, the anesthetic approach must be tailored to each patient’s risk profile rather than applied uniformly. Our patient exhibited no features of mediastinal mass syndrome-there was no orthopnea, positional dyspnea, venous engorgement, or intolerance to the supine position, despite near-complete left lung collapse on imaging. According to the Erdös et al. risk stratification model, which assesses clinical symptoms, positional intolerance, and radiological evidence of compression, the patient was initially considered low risk [6]. Nevertheless, a systematic review risk model incorporating the mass-to-chest ratio, degree of airway and vascular compression, and symptom severity would classify this patient as high risk, warranting vigilant perioperative preparedness [7].

The apparent discrepancy between imaging severity and clinical stability may be explained by the pedunculated and pliable nature of thymolipomas, which allows them to shift within the thoracic cavity and redistribute pressure away from central mediastinal structures in certain positions [2]. Slow-growing mediastinal tumors such as thymolipomas allow for progressive cardiopulmonary adaptation over time. This mobility can mask the physiological impact of compression and render conventional symptom-based risk prediction less reliable.

Given this background, our decision to proceed with controlled induction using muscle relaxation rather than awake intubation was guided by both risk assessment and procedural feasibility. Awake fibreoptic intubation is often considered the safest option for securing the airway in patients with significant mediastinal pathology; however, it presents limitations in cases requiring one-lung ventilation. Placement of a double-lumen tube (DLT) in an awake, spontaneously breathing patient is technically difficult, particularly when airway anatomy is distorted by mass effect. The bulkier and less flexible DLT increases the risk of trauma, coughing, or laryngospasm, each of which could precipitate airway collapse or cardiovascular instability in the presence of a large mediastinal mass [8]. Moreover, repeated unsuccessful attempts at intubation without adequate relaxation may be more hazardous than a controlled, well-prepared induction with full readiness for rescue.

Awake fibreoptic intubation with bronchial blockers could be an alternative strategy but we opted for double lumen tube because bronchial blockers have several limitations in this context. Their placement can be technically challenging in distorted or compressed airways, and they are more prone to displacement once positioned. Lung collapse is often slower and less complete compared to double-lumen tubes, and suctioning of the isolated lung is limited. Additionally, switching between one-lung and two-lung ventilation is cumbersome, which may be problematic when rapid intraoperative changes in ventilation strategy are required.

In our case, additional precautions included denitrogenation in the semi-upright position using pressure-support ventilation, which helped counteract the ventilation-perfusion (V/Q) mismatch caused by lung compression and mediastinal shift. Although such measures may not fully correct hypoxemia, they offer a buffer against rapid desaturation during induction and airway instrumentation [9]. A rigid bronchoscope and an ECMO team were kept on standby to provide immediate airway or circulatory support if collapse occurred. Recent evidence also indicates that maintaining spontaneous ventilation does not uniformly prevent airway obstruction; Gardner et al. reported airway compromise during spontaneous breathing in a patient with a similar mass, suggesting that preserving muscle tone alone does not guarantee patency [10]. Our approach, therefore, prioritised preparedness and control over avoidance, enabling a smooth induction with stable ventilation and oxygenation throughout.

Intraoperatively, PCV-VG was utilised to deliver lung-protective ventilation during one lung ventilation (Tidal volume 4-6 ml per kg predicted body weight, PEEP 5-8cmH_2_O, plateau pressure less than 30cmH_2_O, respiratory rate to maintain normocapnia or mild hypercapnia ) with decelerating flow and consistent pressure, optimising gas exchange while minimising barotrauma. A prolonged expiratory phase was maintained to prevent dynamic air trapping, which in the setting of a large mediastinal mass could increase intrathoracic pressure, reduce venous return, and precipitate cardiovascular collapse [7]. Following tumor excision, gradual re-expansion of the collapsed lung was performed using sustained inflation pressures of 25 cmH₂O for 10-20 seconds, avoiding aggressive manual ventilation to reduce the risk of re-expansion pulmonary edema [11,12].

Effective postoperative pain management is a cornerstone of care following open thoracic procedures, as it facilitates early recovery, improves pulmonary function and reduces the risk of postoperative pulmonary complications. In this case, thoracic epidural analgesia was chosen for its proven efficacy in providing superior dynamic pain relief and promoting optimal respiratory mechanics. Alternative strategies include bilateral erector spinae plane catheters for continuous infusion, patient-controlled analgesia systems, or a multimodal analgesic approach combining regional and systemic techniques to achieve balanced analgesia and minimise opioid requirements.

## Conclusions

This case highlights that even benign anterior mediastinal tumors, such as thymolipoma, can present major anesthetic challenges due to potential airway and cardiovascular compromise. Optimal management of such cases requires meticulous multidisciplinary coordination among the anesthesia, surgical, perfusion, and intensive care teams to ensure perioperative safety and seamless clinical decision making. While traditional teaching favours maintaining spontaneous ventilation, our experience demonstrates that controlled induction with muscle relaxation can be safely employed in carefully selected, clinically stable patients when a well-coordinated, multidisciplinary strategy is in place. The choice of anesthetic induction should be guided by individualized risk stratification rather than dogmatic adherence to a single technique.
